# *PIK3CA* Mutation as Potential Poor Prognostic Marker in Asian Female Breast Cancer Patients Who Received Adjuvant Chemotherapy

**DOI:** 10.3390/curroncol29050236

**Published:** 2022-04-19

**Authors:** Yoon Ah Cho, Seung Yeon Ko, Yong Joon Suh, Sanghwa Kim, Jung Ho Park, Hye-Rim Park, Jinwon Seo, Hyo Geun Choi, Ho Suk Kang, Hyun Lim, Ha Young Park, Mi Jung Kwon

**Affiliations:** 1 Department of Pathology, Hallym University Sacred Heart Hospital, Hallym University College of Medicine, Anyang 14068, Korea; purpleice21@hallym.or.kr (Y.A.C.); hyerim@hallym.or.kr (H.-R.P.); jwseomd@hallym.or.kr (J.S.); 2 Division of Breast and Endocrine Surgery, Catholic Kwandong University International St. Mary’s Hospital, Catholic Kwandong University College of Medicine, Incheon 22711, Korea; syeondr@gmail.com; 3 Division of Breast and Endocrine Surgery, Hallym University Sacred Heart Hospital, Hallym University College of Medicine, Anyang 14068, Korea; nicizm@hallym.or.kr (Y.J.S.); sahakin@hallym.or.kr (S.K.); ringri@hallym.or.kr (J.H.P.); 4 Department of Otorhinolaryngology-Head & Neck Surgery, Hallym University Sacred Heart Hospital, Hallym University College of Medicine, Anyang 14068, Korea; pupen@naver.com; 5 Division of Gastroenterology, Department of Internal Medicine, Hallym University Sacred Heart Hospital, Anyang 14068, Korea; hskang76@hallym.or.kr (H.S.K.); hlim77@hallym.or.kr (H.L.); 6 Department of Pathology, Busan Paik Hospital, Inje University College of Medicine, Busan 48108, Korea; hy08.park@gmail.com

**Keywords:** *PIK3CA* mutation, adjuvant chemotherapy, breast cancer, invasive ductal carcinoma, c-Met, PD-L1, microsatellite instability, mismatch repair proteins

## Abstract

Background: The prognostic relevance of the *PIK3CA* mutation together with PD-L1, c-Met, and mismatch repair deficiency (dMMR) have not been fully investigated in Asian women with breast cancer (BC) who have undergone postoperative adjuvant chemotherapy. Methods: We analyzed *PIK3CA* mutations via peptide nucleic acid (PNA)-mediated real-time PCR assay, PD-L1/c-Met expression via immunohistochemistry (IHC), and microsatellite instability (MSI) status using PCR and IHC, in 191 resected BCs from 2008 to 2011. The Cancer Genome Atlas (TCGA) dataset for the involvement of the *PIK3CA* mutation with PD-L1/c-Met/MMR was explored. Results: The PNA clamp-mediated assay was able to detect the *PIK3CA* mutation in 1% of the mutant population in the cell line validation. Using this method, the *PIK3CA* mutation was found in 78 (49.4%) of 158 samples. c-Met and PD-L1 positivity were identified in 31.4 and 21.8% of samples, respectively, which commonly correlated with high histologic grade and triple-negative subtype. MSI/dMMR was observed in 8.4% of patients, with inconsistency between MMR IHC and the MSI PCR. The *PIK3CA* mutation exhibited a poor prognostic association regarding recurrence-free survival (RFS) in both overall and triple-negative BCs. In subgroup analyses, the *PIK3CA*-mutated tumors showed poorer RFS than the *PIK3CA*-wildtype within the c-Met-positive, MSS, triple-negative, or age onset <50 years subgroups, which showed a similar trend of association in TCGA data. Conclusions: *PIK3CA* mutation together with c-Met or dMMR/MSI status might be relevant to poor prognosis in BC subsets, especially in Asian women.

## 1. Introduction

Breast cancer (BC) represents the most serious causes of malignancy with an estimated 2.26 million cases and cancer-associated mortality with 685,000 deaths for women globally in 2020 [1,2]. In Asia, the incidence rates of BCs are low but have several distinct characteristics from those of western countries; they are rapidly expanding at a higher pace than the western countries, which poses a major clinical challenge [3]. In general, Asian women are more likely to present with a younger age onset (less than 50 years of age; this age range has usually been considered premenopause), and higher grade, stage, or hormone receptor-negative(HR^–^)/triple-negative; and are less likely to have favorable prognosis than women in western countries who have a peak incidence at 60–70 years and HR^+^ tumors [1,3]. Depending on the tumor subtype and stage, major strategies may include surgery, chemotherapy, hormone therapy, targeted therapy, or radiation therapy [4]. In nonmetastatic BC patients, surgical resection is the first treatment of choice and adjuvant therapy can be determined based on the tumor subtype [5]. Adjuvant treatment is an additional therapeutic attempt targeted to improve the time period devoid of disease and survival rate after surgical resection to inhibit micrometastases, by adding standard chemotherapy with or without newer agents [6,7]. Endocrine therapy has been a traditional example for the HR^+^ tumor type; HER2-targeted antibody therapy for HER2^+^ tumor type. A combination with immunotherapy or small-molecule inhibitor therapy may be currently favored as new treatment regimens and options [4]. However, triple-negative BC patients have limited options except for chemotherapy alone [5]. With growing interest in variable treatment options in a new era of personalized medicine, druggable prognostic and predictive biomarkers for BC have drawn particular attention [8]. Recently, the American Society of Clinical Oncology (ASCO) provided a new recommendation for the use of alpelisib, an orally bioavailable, α-specific phosphoinositide 3-kinase (PI3K) inhibitor, in the therapy of postmenopausal patients with HR^+^, HER2^−^, *PIK3CA*-mutated BC [9], which rekindled interest in the *PIK3CA* mutation as an eligible treatment selection biomarker; although this is based on data of the western population [10,11]. However, scarce information is available on the Asian population.

The *PIK3CA* mutation is exemplified as the most frequent molecular abnormality in the PI3K signaling pathway, which is the most recurrently altered in BCs, accounting for 20–40%, that can be therapeutically targeted by small molecules [12]. However, not every patient with *PIK3CA*-mutated BC will gain advantage from PI3K inhibitors; only a 27–29% overall response rate was noted among the *PIK3CA*-mutated BC patients with this treatment [10,11]. This suggests that there may remain other genetic regulators engaged in *PIK3CA* mutation and its clinical outcome.

Oncogenic activation of the PI3K signaling could be mutually influenced by the hepatocyte growth factor (HGF)/MET axis, the programmed death-ligand 1 (PD-L1)/programmed death-1 (PD-1) axis, and microsatellite instability triggered by mismatch repair deficiency (MSI/dMMR), the regulation of which, in our understanding, can contribute to BC treatment [13]. PD-L1/PD-1 axis represents cancer immunotherapy, that utilizes the patient’s immune system to repress tumor cells, and has been drawing attention in the treatment of BC [14]. The binding of PD-L1, an immune inhibitory protein, to PD-1 displayed on the tumor-infiltrating lymphocytes inhibits anticancer immunity and stimulates tumor growth [15]. Both biomarkers of dMMR and MSI-high (MSI-H) have been shown to be reliable predictors for good response to immunotherapy, and are permitted by the United States Food and Drug Administration (FDA) to treat solid tumors with immune checkpoint inhibitors aiming for PD-1, irrespective of tumor origin. c-Met is a receptor tyrosine kinase that upon binding of its ligand, HGF, triggers downstream signaling activities including various vital functions essential in embryologic development and tumor progression [16]. The c-Met/HGF pathway is associated with BC progression and suggests anti-c-Met inhibitors for patients with triple-negative BC [16]. An in vivo study has shown that oncogenic *MET*/*PIK3CA* synergistically induces tumor aggressiveness and chemoresistance [13]. Given that the *PIK3CA* mutation is frequent in BCs, PD-L1, c-Met, and MSI/dMMR might considerably affect BCs, thus, implying that these markers would be tumor behavior-related biomarkers for *PIK3CA*-mutated BC.

In this study, we focused on the *PIK3CA* mutation and its possibly related markers of PD-L1, c-Met, and MSI/dMMR in BCs to determine whether they were relevant to clinical outcomes after adjuvant chemotherapy. We explored whether *PIK3CA* mutation can be involved in the signaling pathways of c-Met, PD-L1, or MSI/MMR in BCs on the basis of TCGA (The Cancer Genome Atlas) data.

## 2. Materials and Methods

### 2.1. Study Design and Patients

Written informed consent was obtained from patients. The Institutional Review Board (IRB No. HALLYM 2019-11-003-001) approved this study. BC specimens were retrospectively extracted from 217 consecutive patients who underwent adjuvant chemotherapy after either modified radical mastectomy or conserving breast surgery and either sentinel lymph node biopsy or axillary lymph node dissection consecutively, from June 2008 to December 2011, at Hallym University Sacred Heart Hospital, Korea. Only patients who were women over 18 years old, diagnosed with primary BCs histologically confirmed as invasive ductal carcinoma (for more homogeneous study enrollment) and were not receiving any neoadjuvant treatment, and whose formalin-fixed, paraffin-embedded (FFPE) blocks were available for analysis were enrolled. All tumors were excised before adjuvant chemotherapy, with all clear resection margins confirmed by frozen biopsy. Finally, 191 patients were included in the study. The median age was 49 years (range 28–80) at the time of diagnosis.

After operation, adjuvant treatments were applied, as was clinically indicated. Of the 191 patients, 101 intravenously received adjuvant 5-fluorouracil 500 mg/m^2^, epirubicin 60 mg/m^2^, and cyclophosphamide 500 mg/m^2^; 45 with doxorubicin 60 mg/m^2^ and cyclophosphamide 600 mg/m^2^; 30 with docetaxel 75 mg/m^2^, doxorubicin 50 mg/m^2^, and cyclophosphamide 500 mg/m^2^; 9 with 5-fluorouracil 500 mg/m^2^, doxorubicin 50 mg/m^2^, and cyclophosphamide 500 mg/m^2^; 3 with 5-fluorouracil 600 mg/m^2^, methotrexate 40 mg/m^2^, and cyclophosphamide 600 mg/m^2^; 2 with paclitaxel 75 mg/m^2^; and 1 with paclitaxel 175 mg/m^2^. Fifty-one patients underwent trastuzumab, and 141 received hormonal therapy.

Clinicopathologic parameters inclusive of age at diagnosis, size, metastasis, and recurrence or death were retrieved from the electric medical charts. Pathologic TNM staging followed the 8th American Joint Committee on Cancer criteria. Histological type and grading were based on the World Health Organization classification.

The average follow-up duration was 106.2 ± 16.3 months. The last follow-up point of time for survival outcome was analyzed until July 2019; 184 patients (181/191, 96.3%) were alive and 22 patients (22/191, 11.5%) had tumor relapse. Among the 22 patients with tumor relapse, 17 survived and 5 died.

### 2.2. Histopathological Analysis

Histologic information and immunohistochemical staining results for estrogen receptor (ER), progesterone receptor (PR), HER2, and Ki-67 were reviewed by two independent board-certified experienced pathologists (YA Cho and MJ Kwon). HR status (ER, PR) was assigned positive by counting the positive tumor nuclei more than 1% [17]. HER2^+^ status was determined by protein overexpression (score of 3) using immunohistochemistry or gene amplification via in situ hybridization [17], which were reviewed from the digital data stored in the hospital’s electric database.

The BC subtypes were defined according to ER, PR, HER2 status, and Ki-67 labeling index, and categorized as follows: luminal A (HR^+^ [ER^+^ and/or PR^+^], HER2^−^, and low Ki-67); luminal B (HR^+^ [ER^+^ and/or PR^+^], and HER2^+^ or HER2^−^, and high Ki-67); HER2-enriched (HR^−^ [ER^−^ and PR^−^] and HER2^+^); and triple-negative (HR^−^ [ER^−^ and PR^−^] and HER2). The cutoff value of high or low Ki-67 was set as 14% [18].

### 2.3. DNA Extraction and PIK3CA Mutation Analysis

Genomic DNA extraction was conducted from two slides of 5 µm thick FFPE sections through the Maxwell 16 FFPE Purification Kit for DNA (Promega, Madison, WI, USA). Their concentration and purity were examined by a NanoDrop ND-1000 spectrophotometer (NanoDrop Technologies, Wilmington, NC, USA). The average concentration of obtained DNA was 45.27 ng/μL (range, 19.50–146.70 ng/μL), and the estimated 260/280 purity was from 1.88 to 3.99. The DNA samples were stored at −20 °C unless used promptly.

Alteration of *PIK3CA* variants were identified using the PNAClamp PIK3CA Mutation Detection kit (Panagene, Daejeon, Korea), which exploits modified PCR technology applying optimized peptide nucleic acid (PNA) probes that firmly bind to wild-type DNA templates (Supplementary Table S1). Those firm bindings to the wild-type DNA templates led to no amplification of the wild-type DNA template during polymerase chain reaction (PCR), whereas the mutated DNA templates were processed for multiplication. All detailed procedures and calculation methods were used as previously described [19,20].

### 2.4. Immunohistochemistry and MSI/MMR Analysis

Immunohistochemical staining, except for PD-L1, was assessed on 4 μm thick tissue sections of microarray with two 3.0 mm tumor cores employing BenchMark XT automated immunostainer system (Ventana Medical Systems, Inc., Tucson, AZ, USA), as per the manufacturers’ manuals. The primary antibody used was anti-c-Met (rabbit polyclonal, pre-diluted; Ventana Medical System) for 40 min at 37 °C, then a secondary antibody of Universal HRP Multimer (Ventana Medical System) was used for 8 min at 37 °C. Then, the sections were incubated with chromogen diaminobenzidine (ultraView Universal DAB Kit, Ventana Medical System) and counterstained with hematoxylin. The following primary antibodies as for MMR proteins were applied: anti-MLH1 (pre-diluted; Ventana Medical Systems), anti-MLH2 (1:300; Cell Marque, Rocklin, CA, USA), anti-PMS2 (pre-diluted; Ventana Medical Systems), and anti-MSH6 (1:200; Cell Marque). PD-L1 staining was carried out by the FDA-approved PD-L1 22C3 pharmDx kit (Dako North America Inc., Carpinteria, CA, USA) on the Dako AutostainerLink 48, according to the manufacturer’s instructions [21].

For PD-L1, the slides were assigned based on the percentage of positive cells separated by the number of fields to calculate the mean value for each individual case, defined at 200× magnification. The PD-L1 combined positive score was calculated with respect to the ratio of PD-L1-positive cells (tumor or immune cells) to the total number of tumor cells × 100, and was categorized into positive (≥1) or negative (<1).

Interpretation of c-Met expression based on adjusted scoring value described in clinical trials regarding the MET inhibitor was adapted as follows [22]: 0, no staining or staining intensity in <50% tumor cells; 1+, weak-to-moderate staining intensity in >50% tumor cells; 2+, moderate-to-strong staining intensity in >50% of tumor cells; 3+, strong staining intensity in >50% tumor cells in terms of membranous and/or cytoplasmic staining. Scores of 2+ or 3+ were considered as c-Met-positive, and those of 0 or 1+ as c-Met-negative.

MSI/MMR status was determined depending on loss of expression of MMR proteins and/or by analysis of melting peak using real-time PCR on five quasi-monomorphic mononucleotide repeat markers such as NR21, NR24, NR27, BAT25, and BAT26, assessing a U-Top Microsatellite Instability Detection Kit (Seasun Biomaterials Inc., Daejeon, Korea) for FFPE normal and tumor tissues. MSI-low (MSI-L) and MSI-H were divided when allelic size variation occurred in either one or two of the five mononucleotide markers, respectively. The MSI/dMMR was assigned as either loss of expression of one or more MMR proteins or allelic size variation in one or more of the five quasi-monomorphic markers, which was conducted by PCR; microsatellite-stable/proficient-MMR (MSS/pMMR) as both intact expression of MMR proteins and absence of any MSI [23].

### 2.5. TCGA Dataset Analysis for PD-L1/c-Met/MMR Related to PIK3CA Mutation

Mutational status of *PIK3CA* gene and mRNA expression profile of 6 genes (*PD-L1*, *MET*, *MLH1*, *MSH2*, *MSH6*, and *PMS2*) was downloaded from the “Breast Invasive Carcinoma” dataset of the TCGA (http://cancergenome.nih.gov/abouttcga (accessed on 27 January 2022)) in cBioPortal (https://www.cbioportal.org/ (accessed on 27 January 2022)). Of the 1108 samples included in the dataset, 978 samples which had available mutational status and expression profiles were used. Regarding the mutational status of *PIK3CA*, 978 samples were categorized as wild-type (*n* = 660) and mutant (*n* = 318). We compared expressional differences of 6 genes for *PIK3CA* mutational status for statistical analysis calculated by Mann–Whitney U test.

### 2.6. Statistical Analysis

The categorical variables were determined by means of the chi-squared test or two-sided Fisher’s exact test. Survival curves were compared assessing Kaplan–Meier estimates and the log-rank test. Overall survival (OS) was determined as the interval time from the day of surgery to death of any cause or last follow-up; recurrence-free survival (RFS) was indicated as the gap of time from the day of surgery to the day occurring relapse of tumor, death of any cause, or the last follow-up. Hazard ratios were obtained with Cox regression for the univariate and multivariate analyses of OS and RFS, and verified to fulfill assumptions for proportional hazards. Statistical analyses were conducted with SPSS for Windows version 21.0 (SPSS Inc., Chicago, IL, USA). Statistical significance was considered a two-sided *p*-value of <0.05.

## 3. Results

### 3.1. Validation of the Assay for PIK3CA Mutation

**Figure 1:**
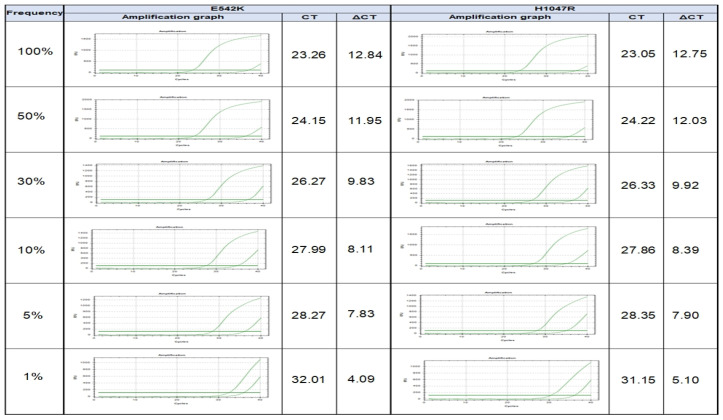
Validation of detection rate of *PIK3CA* mutation in serially diluted cell line experiment from 100, 50, 20, 10, 5, to 1% of *PIK3CA*-mutant alleles.

To investigate the detection capability of the method (PNA clamp real-time PCR), PIK3CA-mutated (A549 cell line) DNA was serially diluted to generate samples containing 100, 50, 20, 10, 5, and 1% of PIK3CA-mutant alleles (E542K and H1047R, respectively), which were subjected to PNA clamp real-time PCR to independently determine each detection rate of the diluted PIK3CA-mutant alleles. The ΔCt1 values of the 100-, 5-, 2-, 10-, 5-, and 1%-mutant samples were 12.84, 11.95, 9.83, 8.11, 7.83, and 4.09 for E542K-mutant alleles and 12.75, 12.03, 9.92, 8.39, 7.90, and 5.10 for H1047R-mutant alleles, respectively. According to a ΔCt1 cutoff point of ≥2.0, the PNA clamp real-time PCR assay was capable of identifying the PIK3CA mutation in a 1%-mutant population (Figure 1).

### 3.2. PIK3CA Mutation and TCGA Dataset Analysis

Through the TCGA dataset analysis for PIK3CA mutation and mRNA expression profile of six genes (PD-L1, MET, MLH1, PMS2, MSH2, and MSH6), we identified that PIK3CA mutations were potentially able to be connected with the signaling pathways of MET, MSH2, and MSH6 in BCs based on TCGA data (Supplementary Table S2).

The PIK3CA mutation was evaluated in 191 cases, of which 158 cases had available PIK3CA mutational results: 78 (49.4%) were PIK3CA-mutated and 80 (50.6%) were PIK3CA-wildtype. PIK3CA hotspot mutations (H1047, E542, E545) were found in 48.1% of BC patients (76/158), with only two cases (1.3%) harboring non-hotspot mutation (C420). The most frequent alteration of the PIK3CA gene was H1047 (34.2%). Thirteen patients (8.2%) carried two or more concurrent mutations in PIK3CA.

The frequency of PIK3CA mutations was 57.1% (16/28) in the luminal A subtype, 45.7% (37/81) in the luminal B subtype, 37.5% in the HER2-enriched subtype, and 52.8% in the triple-negative subtype, of which the differences showed no statistical significance (*p* = 0.406). There were no associations of the PIK3CA mutation with clinical or pathological characteristics (Table 1).

### 3.3. c-Met, PD-L1, and MMR/MSI

c-Met, PD-L1 expression and MSI/MMR status were available for 170 samples, 169 samples, and 167 cases, respectively. The positive rates of PD-L1, c-Met, and MSI/dMMR were demonstrated in 37 (21.8%), 53 (31.4%), and 14 (8.4%) of the cases examined, respectively. PIK3CA mutation was most commonly expressed together with c-Met (36.8%), followed by PD-L1 (20.6%) and MSI/dMMR (11.9%).

PD-L1 overexpression was significantly correlated with c-Met positivity (*p* = 0.033). Both PD-L1 and c-Met expressions showed association with high histologic grade (*p* < 0.001 and *p* < 0.001, respectively), ER^−^ (*p* = 0.007 and *p* < 0.001, respectively), PR^−^ (*p* = 0.022 and *p* = 0.001, respectively), and BC subtype (*p* = 0.028 and *p* = 0.001, respectively).

We observed 14 cases (8.4%) that showed dMMR: immunohistochemically, complete loss of expression of MSH2/MSH6 (*n* = 7), MLH1/MSH2/PMS2/MSH6 (*n* = 6), and MSH6 (*n* = 1), of which results more closely related to MSH2 and MSH6 considerably keep in line with the TCGA data analysis. Those dMMR cases showed three cases of MSI-L, two of MSI-H, and nine of MSS using real-time PCR. Among the five quasi-monomorphic markers, BAT26 marker was the most commonly exhibited MSI (*n* = 4), next with the NR24 (*n* = 2) and NR21 (*n* = 1) markers in sequence.

### 3.4. Prognostic Implications

**Figure 2:**
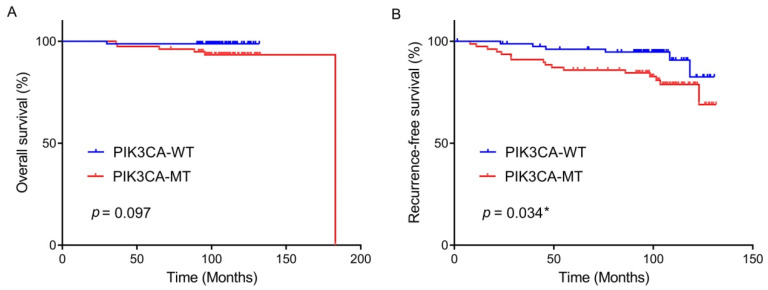
Overall survival rate (**A**) and recurrence-free survival rate (**B**) by *PIK3CA* mutational status in the patients with breast cancer who underwent curative surgery and adjuvant chemotherapy. * statistically significant, *p* value < 0.05.

Kaplan–Meier survival analysis was attempted to estimate whether PIK3CA mutation, c-Met/PD-L1 expression, and MSI/dMMR were associated with OS or RFS in patients with BC who received adjuvant chemotherapy following surgery. Kaplan–Meier curves displayed that the PIK3CA-mutated BCs had a tendency for unfavorable OS when compared to the PIK3CA-wildtype; however, it reached no statistical significance (*p* = 0.097, Figure 2A). Patients with PIK3CA-mutated tumors showed a worse RFS rate than those with PIK3CA-wildtype tumors (mean 114 vs. 124 months) (*p* = 0.034, Figure 2B).

Patients with c-Met-expressed BC had a shorter RFS than those with c-Met-negative tumors (mean 111 vs. 123 months) (*p* = 0.047); there was no significant difference in OS (*p* = 0.788). The OS and RFS between patients with PD-L1-positive and those with PD-L1-negative showed no statistical differences (*p* = 0.873 and *p* = 0.241, respectively); between patients with MSI/dMMR and those with MSS/pMMR (*p* = 0.224 and *p* = 0.658, respectively).

To explore prognostic factors for OS and RFS in patients with BCs, univariate and multivariate Cox proportional hazard regression methods were analyzed (Table 2), which also supported the relevance of PIK3CA mutation with worse RFS, but not with OS. The univariate analysis revealed no PD-L1, c-Met expression, or MSI/dMMR influence on OS or RFS, although the trend toward a poor RFS in c-Met expressed tumors showed borderline statistical significance (*p* = 0.052). Lymphovascular invasion significantly affected OS (*p* = 0.011). From the multivariate analysis, PIK3CA mutation was proved to be an independent poor prognostic factor related to RFS (hazard ratio 3.543, 95% CI 1.047–11.988, *p* = 0.042).

**Figure 3:**
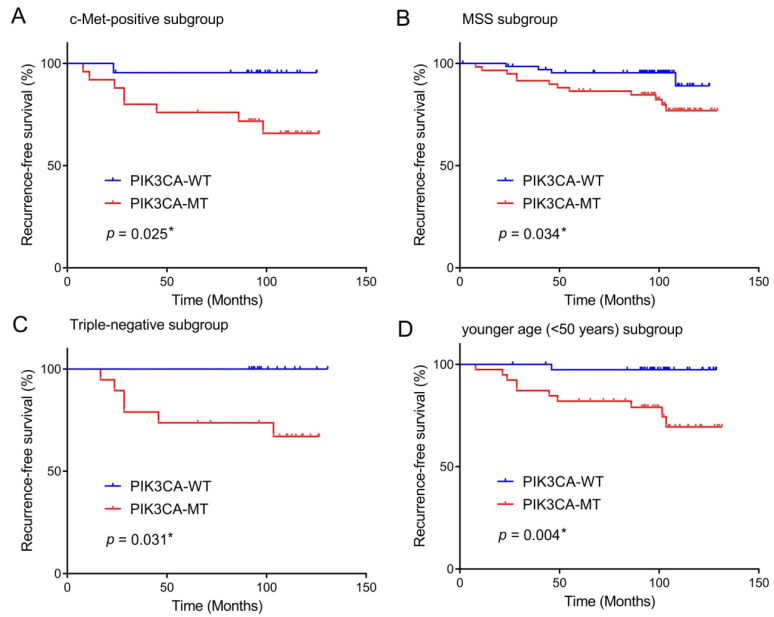
Prognostic impact of *PIK3CA* mutation in breast cancers according to subgroups. The presence of *PIK3CA* mutation predicts unfavorable recurrence-free survival of patients with c-Met-positive tumors (**A**), MSS tumors (**B**), triple-negative subtype (**C**), age younger than 50 years old (**D**). * statistically significant, *p* value < 0.05.

In the subgroup analyses, the PIK3CA mutation was concerned with a worse RFS rate in the subgroups of patients showing c-Met-positive tumors (*p* = 0.025), MSS tumors (*p* = 0.034), triple-negative subtype (*p* = 0.031), and younger age onset <50 years (*p* = 0.004) (Figure 3). There was not any significant difference for PIK3CA mutation in the RFS of PD-L1-positive BCs (*p* = 0.112).

## 4. Discussion

In the current study, we identified a significant correlation of the *PIK3CA* mutation with the signaling pathways of c-Met and dMMR in BCs, based on the TCGA database, as well as the poor prognostic role of the *PIK3CA* mutation with c-Met and MSI/MMR expression in BCs. The *PIK3CA* mutation, comprising approximately 50%, was a poor prognostic factor for worse RFS in the patient cohort that received adjuvant chemotherapy following surgery, particularly for c-Met-positive, MSS, triple-negative, or younger age onset <50 years subtypes.

Since the *PIK3CA* mutation is associated with both the efficacy of PI3K inhibitor and other endocrine or targeted therapy [24], it is of great clinical importance to precisely demonstrate the *PIK3CA* mutational status and prognosticate the therapeutic effects in BC. In this study, the *PIK3CA* mutation revealed in 49.4% of all the BCs, with H1047 ranking the highest substitution using a PNA-based clamping approach to identify a *PIK3CA*-mutant proportion as low as 1% in the cell line experiment, a finding that validates the highly sensitive detection method. This frequency of *PIK3CA* mutation is within a wide range (25.6–59.8%) of previous studies worldwide, and the most common genotype, H1047, was in concordance [25,26,27,28], and was closely compatible with 46.5% of that recently described in the corresponding Far East Asian area using next-generation sequencing (NGS) [27]. Recently, NGS was recommended by ASCO for the detection of *PIK3CA* mutations for treatment eligibility for alpelisib among patients with luminal subtype BC [9]. However, this method is too expensive to be readily available to much of the world. Nearly two-thirds of new BC cases and deaths are confronted in less developed countries these days [2]. We noted the lack of particular clinical or demographic characteristics linked to the presence of the *PIK3CA* mutation among the patients, which was consistent with other studies [29]; any subset of clinicopathological factors are unlikely to indicate a certain group of patients expected to carry the *PIK3CA* mutation, indicating that all BC patients should be tested for the *PIK3CA* gene in order to detect the mutation. Alternatively, this requires economic considerations of cost-effective tests.

Resistance to chemotherapy and poor prognosis could be mediated by the activation of the PI3K pathway, which allows a survival signaling for withstanding anticarcinogenic agents and enhancing cancer stem cell characteristics [30,31]. In BCs, the *PIK3CA* mutation has been shown to correlate with resistance to paclitaxel [32] or anti-HER2 adjuvant therapy [33,34]. Although controversial, considerable literature has shown a correlation of *PIK3CA* mutation with untoward clinical outcomes [12,27,29,35,36,37,38,39,40,41]. However, the prognostic usefulness of the *PIK3CA* mutation after adjuvant chemotherapy has been suggested in limited BC subtypes and has not been fully elucidated [11,24], especially in Asian populations. A phase III clinical trial demonstrated the *PIK3CA* mutation as an unfavorable prognostic indicator highly relevant to standard adjuvant chemotherapeutic outcome in HR^+^/HER2^−^ metastatic BCs [11]. One recent meta-analysis was also compatible in the finding that *PIK3CA* mutation may serve as a crucial prognostic predictor for a gloomy prognosis of HR^+^/HER2^−^ BCs, not only in PI3K-inhibitor therapy groups but also in non-PI3K-inhibiting therapy groups [24]. However, those studies mainly focused on the western population, which is comparatively different from BCs in Asian women [1]. In our cohort who received postoperative adjuvant chemotherapy, a strong association was found between *PIK3CA* mutation and worse RFS, but not with OS; the *PIK3CA* mutation may be an independent poor prognostic factor that can be used to predict worse RFS rates in Korean patients after adjuvant chemotherapy. We also found the considerable frequency of *PIK3CA* mutation all across BC subtypes, the luminal subtype (HR^+^/HER2^−^; 48.6%), HER2-enriched (39.5%), and triple-negative (57.6%) tumors in BCs overall with an average age of 49 years at diagnosis, which supported the apparently different biologic impact of *PIK3CA* mutation on specific cancer subtypes as previously described [11,33]. Interestingly, the subgroup analyses exhibited a positive prognostic correlation of the *PIK3CA* mutation in the triple-negative subtype, which may provide further prognostic relevance for the *PIK3CA* mutation, reflecting the epidemiologic characteristics of Asian BCs. The lack of prognostic relevance with OS in our analyses might be explained by the survival benefit achieved from adjuvant treatment, which is consistent with a steady decline in the mortality rate from BC owing to the development of adjuvant treatment modalities in the last two decades [42].

There are few available data regarding the prognostic link between *PIK3CA* mutation and PD-L1/c-Met/MSI status for BCs. In this study, using FDA-approved PD-L1 22C3 assay and clinical trial-relevant c-Met scoring criteria, PD-L1 and c-Met positivity were identified in 21.8 and 31.4% of cases, respectively, which commonly correlated with high histologic grade, HR^−^ (ER^−^ and PR^−^), triple-negative subtype. Similar to our results, PD-L1, overexpressed in 23.4% of BCs, has been correlated with higher stage/tumor grade/Ki-67, older age, and ER negativity; also, it has been described as a poor prognostic factor of BC [43]. The PD-L1 mRNA expression level has been associated with triple-negative BC subtype [44]. Because it has been suggested that the combined assessment of c-Met expression and *PIK3CA* mutational status would be biomarkers for patient stratification unlikely to yield resistance to paclitaxel or anti-HER2 targeted therapy [13,16], this indicates that c-Met along with *PIK3CA* mutation appear to closely associate with unfavorable clinical outcome. In subgroup analyses, tumors with *PIK3CA* mutation exhibited worse RFS than those with *PIK3CA*-wildtype within the c-Met-positive or MSS subgroups, which may imply the indirect impact of *PIK3CA* mutation with c-Met or MMR status and might ultimately lead to poor prognosis; this seems to be in keeping with our TCGA data analysis. Of the six genes examined, our TCGA dataset analysis results showed the association of *MET*, *MSH2*, and *MSH6* gene expression with *PIK3CA* mutation in BCs. The present study might propose that a combined analyses of *PIK3CA* mutational status together with c-Met or MMR status, and an intrinsic subtype may help to prognostically estimate RFS outcome after adjuvant therapy following surgery, especially in Asian women, with probably high risk for earlier onset under 50 years of age and triple-negative BC [1,3]. Conversely, a *PIK3CA* inhibitor might be a potential therapeutic strategy for preventing relapse in *PIK3CA* mutated, c-Met-positive, or MSS BCs after adjuvant therapy. Since c-Met, PD-L1, MMR protein expression may be readily implemented in an immunohistochemical processing system, the relevant survival results shown in the study may imply that those markers, together with *PIK3CA* mutation, may be used as prognostic markers in BC.

We demonstrated the low incidence of MSI/dMMR in 8.4% of the patients examined, which alone had no prognostic association. MSI/dMMR has been rarely reported in a broad range of 7.2–30% in overall BCs [45,46]. We noted the BAT26 marker to be the most commonly exhibited MSI, a finding that was rarely mentioned in BCs [46]. Inconsistencies were noted in the results (8.4 vs. 3.0%) of the MMR and the MSI approach in the present study. In addition, MSH2 and MSH6 seem to be more likely involved in *PIK3CA* mutation in BCs, rather than MLH1 or PMS2, suggesting MSH2 and MSH6 are more reliable markers in BCs. These may explain the possible association between *PIK3CA* mutation and limited MMR proteins (*MSH2* and *MSH6* gene expression) in BCs of the TCGA dataset analysis. This phenomenon has also been described in the other BC cohorts [46], where it was explained that the accumulation of identifiable MSI occurs as a late stage event secondarily after impaired functioning of MMR proteins [46]. Since dMMR or MSI-H are represented as beneficial predictors for responsiveness to immunotherapy [46], any cases with either dMMR or MSI-H were considered as MSI/dMMR tumors in our analyses. We found no correlation of survival with MSI/dMMR or c-Met/PD-L1 expression alone, which was in keeping with a recent study where there was a lack of any correlation between MSI/dMMR and clinicopathological features, PD-L1 expression, or survival in triple-negative BCs [46]. This might be because BC is less immunogenic [47]. In colorectal cancers, the most extensively studied for links between antitumor immunity and mismatch repair systems, the *TP53* mutation has been shown to suppress antitumor immunity that may contribute to a cancer-promoting state [48]. Other than *PIK3CA* mutation, *TP53* mutations are the second most (24.7–33.9%) common in breast cancers, with the frequency of their co-mutations being 8.7–12.8% [28,49]. The collaboration of the *PIK3CA* mutation (H1047R) with the *TP53* mutation has initiated mammary tumorigenesis in animal models [50]. The patients with *TP53*-*PIK3CA* co-mutation have shown worse clinical outcome compared to others [49]. In part, the discrepancy might be related to the possible confounders that were not examined in this study. Although uncommon, MSI/dMMR provided only limited insight, it is worthy of note that the two MSI-H cases in the present study carried multiple alterations of the *PIK3CA* gene with high nuclear grade and histologic grade 3, without recurrence or death during the study period. Such cases may emphasize the trend of MSI toward favorable prognosis despite advanced disease with aggressive histologic features.

## 5. Conclusions

The limitations of the present study comprise the single-center retrospective implementation with a relatively small sample size and potentially unmeasured confounders such as the lack of information of *TP53* mutation, body mass index indicating obesity, or numbers of childbearing (that may be factors involved in increased risk for breast cancers). Nonetheless, the findings raise certain intriguing points. The *PIK3CA* mutation exhibited unfavorable prognostic significance in patients with both overall and triple-negative BCs, indicating the potential of *PIK3CA* mutation with c-Met or MSI/MMR as a detailed prognostic marker in BC subsets, especially in Asian women. The findings from the current study may pave way for further prospective investigations and may be clinically relevant in the future for the personalized management of BC in Asian women, both for treatment and follow-up strategies.

## Figures and Tables

**Table 1:** Clinicopathologic correlations of *PIK3CA* mutation, PD-L1 and c-Met expression, and MSI/dMMR status.

Characteristic	*PIK3CA*		*p*	PD-L1 Expression		*p*	c-Met Expression		*p*
	MT	WT		Positive	Negative		Positive	Negative	
	*n* (%)	*n* (%)		*n* (%)	*n* (%)		*n* (%)	*n* (%)	
Age (years)			1.000			0.703			0.609
<50	39 (50.0)	40 (50.0)		18 (48.6)	60 (45.1)		26 (49.1)	52 (44.8)	
≥50	39 (50.0)	40 (50.0)		19 (51.4)	73 (54.9)		27 (50.9)	64 (55.2)	
pT category			0.874			0.265			0.410
pT1	35 (44.9)	37 (46.3)		15 (40.5)	70 (52.6)		24 (45.3)	61 (52.6)	
pT2–pT3	43 (55.1)	43 (53.7)		22 (59.5)	63 (47.4)		29 (54.7)	55 (47.4)	
pN category			0.752			0.852			0.407
pN0	43 (55.1)	42 (52.5)		22 (59.5)	76 (57.1)		33 (62.3)	64 (55.2)	
pN ≥ 1	35 (44.9)	38 (47.5)		15 (40.5)	57 (42.9)		20 (37.7)	52 (44.8)	
Histologic grade			0.527			<0.001 *			<0.001 *
I–II	45 (57.7)	42 (52.5)		10 (27.0)	89 (66.9)		19 (35.8)	80 (69.0)	
III	33 (42.3)	38 (47.5)		27 (73.0)	44 (33.1)		34 (64.2)	36 (31.0)	
LVI			0.286			1.000			0.335
Absent	60 (76.9)	55 (68.8)		28 (75.7)	100 (75.2)		43 (81.1)	85 (73.3)	
Present	18 (23.1)	25 (31.2)		9 (24.3)	33 (24.8)		10 (18.9)	31 (26.7)	
ER			1.000			0.007 *			<0.001 *
Negative	25 (32.1)	26 (32.5)		19 (51.4)	37 (27.8)		29 (54.7)	26 (22.4)	
Positive	53 (67.9)	54 (67.5)		18 (48.6)	96 (72.2)		24 (45.3)	90 (77.6)	
PR			1.000			0.022 *			0.001 *
Negative	28 (35.9)	29 (36.3)		18 (48.6)	38 (28.6)		27 (50.9)	28 (24.1)	
Positive	50 (64.1)	51 (63.7)		19 (51.4)	95 (71.4)		26 (49.1)	88 (75.9)	
HER2			0.154			0.531			0.573
Negative	61 (78.2)	54 (67.5)		29 (78.4)	95 (71.4)		41 (77.4)	84 (72.4)	
Positive	17 (21.8)	26 (32.5)		8 (21.6)	38 (28.6)		12 (22.6)	32 (27.6)	
Subtype			0.406			0.028 *			0.001 *
Luminal A	16 (20.5)	12 (15.0)		3 (8.1)	24 (18.0)		7 (13.2)	21 (18.1)	
Luminal B	37 (47.4)	44 (55.0)		18 (48.6)	75 (56.4)		20 (37.7)	72 (62.1)	
HER2-enriched	6 (7.7)	10 (12.5)		3 (8.1)	15 (11.3)		7 (13.2)	10 (8.6)	
Triple-negative	19 (24.4)	14 (17.5)		13 (35.2)	19 (14.3)		19 (35.9)	13 (11.2)	
MSI/MMR			0.196			0.736			0.926
MSS/pMMR	59 (89.4)	68 (95.8)		34 (94.4)	118 (91.5)		49 (92.5)	104 (92.9)	
MSI/dMMR	7 (10.6)	3 (4.2)		2 (5.6)	11 (8.5)		4 (7.5)	8 (7.1)	
*PIK3CA* status			-			0.685			0.591
WT	-	-		17 (54.8)	53 (49.5)		22 (46.8)	48 (52.7)	
MT	-	-		14 (45.2)	54 (50.5)		25 (53.2)	43 (47.3)	
PD-L1			0.685			-			0.033 *
Negative	54 (79.4)	53 (75.7)		-	-		36 (67.9)	95 (82.6)	
Positive	14 (20.6)	17 (24.3)		-	-		17 (32.1)	20 (17.4)	
c-Met			0.591			0.033 *			-
Negative	43 (63.2)	48 (68.6)		20 (54.1)	95 (72.5)		-	-	
Positive	25 (36.8)	22 (31.4)		17 (45.9)	36 (27.5)		-	-	

Abbreviations: PD-L1—programmed death ligand-1; MT—mutated; WT—wild type; LVI—lymphovascular invasion; ER—estrogen receptor; PR—progesterone receptor; MSI—microsatellite instability; pMMR—patent mismatch repair; dMMR—deficient mismatch repair. * statistically significant, *p* value < 0.05.

**Table 2:** Univariate and multivariate analyses of overall survival and recurrence-free survival of patients with breast cancers.

Characteristic	Overall Survival	*p*	Recurrence-Free Survival	*p*
	Hazard Raio (95% CI)		Hazard Raio (95% CI)	
Univariate analysis				
Age (y) (<50 vs. ≥50)	0.892 (0.180–4.420)	0.889	0.958 (0.413–2.222)	0.921
Histologic grade (I vs. II–III)	1.423 (0.287–7.052)	0.666	0.775 (0.325–1.849)	0.565
LVI (absent vs. present)	16.00 (1.869–137.04)	0.011 *	1.452 (0.592–3.564)	0.416
pT1 vs. pT2–pT3	5.082 (0.594–43.498)	0.138	0.863 (0.372–2.000)	0.731
LNM (absent vs. present)	6.757 (0.789–57.843)	0.081	1.607 (0.693–3.731)	0.269
ER (negative vs. positive)	0.464 (0.094–2.300)	0.347	1.015 (0.414–2.491)	0.974
PR (negative vs. positive)	0.523 (0.106–2.593)	0.428	0.545 (0.235–1.264)	0.157
HER2 (negative vs. positive)	2.782 (0.561–13.784)	0.210	0.597 (0.202–1.764)	0.350
*PIK3CA* (WT vs. MT)	5.116 (0.598–43.797)	0.136	2.662 (1.041–6.810)	0.041 *
PD-L1 (negative vs. positive)	1.203 (0.125–11.568)	0.873	0.426 (0.098–1.853)	0.255
c-Met (negative vs. positive)	0.734 (0.076–7.054)	0.789	1.712 (0.996–2.942)	0.052
MSS/pMMR vs. MSI/dMMR	3.988 (0.415–38.349)	0.231	0.703 (0.093–5.302)	0.732
Multivariate analysis				
Age (y) (<50 vs. ≥50)	0.819 (0.084–7.989)	0.863	0.514 (0.177–1.496)	0.222
Histologic grade (I vs. II–III)	0.704 (0.044–11.291)	0.805	0.865 (0.201–3.716)	0.846
LVI (absent vs. present)	10.786 (0.418–278.281)	0.152	1.879 (0.534–6.611)	0.326
pT1 vs. pT2–pT3	2.577 (0.120–55.164)	0.545	0.596 (0.195–1.823)	0.365
LNM (absent vs. present)	2.753 (0.131–57.835)	0.514	0.626 (0.190–2.065)	0.442
ER (negative vs. positive)	0.194 (0.001–33.231)	0.532	14.086 (1.471–134.862)	0.022 *
PR (negative vs. positive)	2.262 (0.014–378.806)	0.755	0.056 (0.008–0.420)	0.005 *
HER2 (negative vs. positive)	2.852 (0.241–33.717)	0.406	0.294 (0.052–1.651)	0.164
*PIK3CA* (WT vs. MT)	7.758 (0.400–150.40)	0.176	3.543 (1.047–11.988)	0.042 *
PD-L1 (negative vs. positive)	2.477 (0.126–48.792)	0.551	0.279 (0.047–1.675)	0.163
c-Met (negative vs. positive)	0.360 (0.011–12.246)	0.570	1.956 (0.611–6.262)	0.259
MSS/pMMR vs. MSI/dMMR	5.821 (0.229–147.70)	0.286	0.906 (0.105–7.855)	0.929

Abbreviations: CI—confidence interval; LVI—lymphovascular invasion; LNM—lymph node metastasis; ER—estrogen receptor; PR—progesterone receptor; PD-L1—programmed death ligand-1; MSI—microsatellite instability; dMMR—deficient mismatch repair. * statistically significant, *p* value < 0.05.

## Data Availability

The data used to support the findings of this study are available from the corresponding author upon request.

## Supplementary Materials

The following supporting information can be downloaded at: https://www.mdpi.com/article/10.3390/curroncol29050236/s1, Table S1: The PNA-mediated clamping assay detects mutations of *PIK3CA* gene; Table S2: TCGA data analysis between *PIK3CA* mutation and expressions of *PD-L1*, *MET*, and mismatch repair (*MLH1*, *MSH2*, *MSH6* and *PMS2*) genes.
