# Supplementary material for: PIK3CA Mutation as Potential Poor Prognostic Marker in Asian Female Breast Cancer Patients Who Received Adjuvant Chemotherapy
Source: Curr Oncol. 2022 Apr 19;29(5):2895–908. doi: 10.3390/curroncol29050236 (PMC9140087; doi:10.3390/curroncol29050236)
Supplement: Supplementary file 1 [file curroncol-29-00236-s001.zip › curroncol-1656685-supplementary.pdf]

Supplementary Tables

Supplementary Table S1. The PNA-mediated clamping assay detects mutations of *PIK3CA* gene

No.	Mutation	Exon	Base change
1	E542G	9	1625A>G
2	E542V	9	1625A>T
3	E542K	9	1624G>A
4	E545K	9	1633G>A
5	E545G	9	1634A>G
6	E545D	9	1635G>T
7	Q546E	9	1636C>G
8	Q546K	9	1636C>A
9	Q546P	9	1637A>C
10	Q546R	9	1637A>G
11	E545A	9	1634A>C
12	H1047Y	20	3139C>T
13	H1047L	20	3140A>T
14	H1047R	20	3140A>G
15	C420R	7	1258T>C

Supplementary Table S2. TCGA data analysis between *PIK3CA* mutation and expressions of *PD-L1*, *MET*, and mismatch repair (*MLH1*, *MSH2*, *MSH6* and *PMS2*) genes.

	PIK3CA status		
	Mutant	Wild type	
Genes*	(N=318)	(N=660)	*P*-value
PD-L1	20.1	19.84	0.8208
MET	303.42	392.55	<0.001
MLH1	821.14	799.09	0.2653
MSH2	838.33	955.47	<0.001
MSH6	1580.17	1812.73	<0.001
PMS2	736.86	744.05	0.1635

* Median of expression values (RSEM)
